# Developing Antibiofilm Fibrillar Scaffold with Intrinsic Capacity to Produce Silver Nanoparticles

**DOI:** 10.3390/ijms232315378

**Published:** 2022-12-06

**Authors:** Giovanna Pitarresi, Giuseppe Barberi, Fabio Salvatore Palumbo, Domenico Schillaci, Calogero Fiorica, Valentina Catania, Serena Indelicato, David Bongiorno, Giuseppina Biscari, Gaetano Giammona

**Affiliations:** 1 Department of Biological, Chemical and Pharmaceutical Sciences and Technologies (STEBICEF), University of Palermo, Via Archirafi 32, 90123 Palermo, Italy; 2 Department of Earth and Marine Sciences (DiSTeM), University of Palermo, Viale delle Scienze Ed. 16, 90128 Palermo, Italy

**Keywords:** electrospinning, catechol groups, silver nanoparticles, anti *Pseudomonas aeruginosa* activity

## Abstract

The development of biomedical systems with antimicrobial and antibiofilm properties is a difficult medical task for preventing bacterial adhesion and growth on implanted devices. In this work, a fibrillar scaffold was produced by electrospinning a polymeric organic dispersion of polylactic acid (PLA) and poly(α,β-(N-(3,4-dihydroxyphenethyl)-L-aspartamide-co-α,β-N-(2-hydroxyethyl)-L-aspartamide) (PDAEA). The pendant catechol groups of PDAEA were used to reduce silver ions in situ and produce silver nanoparticles onto the surface of the electrospun fibers through a simple and reproducible procedure. The morphological and physicochemical characterization of the obtained scaffolds were studied and compared with virgin PLA electrospun sample. Antibiofilm properties against *Pseudomonas aeruginosa*, used as a biofilm-forming pathogen model, were also studied on planar and tubular scaffolds. These last were fabricated as a proof of concept to demonstrate the possibility to obtain antimicrobial devices with different shape and dimension potentially useful for different biomedical applications. The results suggest a promising approach for the development of antimicrobial and antibiofilm scaffolds.

## 1. Introduction

The development of bacterial infections raises great concern about the increasing antibiotic-resistance developed by multidrug-resistant bacteria (MDR) [1,2]. Bacterial proliferation and biofilm formation in implanted medical devices represent, today, problems of relevant clinical importance [3,4]. Most implant-associated infections are caused by bacterial contamination of the wound occurring during surgical procedures: bacteria can settle on both the surface of the implanted device and the damaged tissue, forming biofilms through which bacteria evade host defenses and antimicrobial treatment [5,6,7,8]. The development of biomedical systems with antimicrobial and antibiofilm properties is a promising strategy to prevent bacterial adhesion and growth.

From this point of view, non-specific therapeutic approaches are often proposed [9,10,11,12]. Metal nanoparticles and, especially, silver nanoparticles (AgNPs) have broad-spectrum antimicrobial properties and are active against bacteria, fungi, viruses, and other microorganisms [13,14]. AgNPs are able to eradicate even MDR bacteria due to the capability to act through several mechanisms such as interaction with the bacterial membrane [15,16], the release of Ag^+^ ions [17,18], and the production of reactive oxygen species [17].

Several procedures (chemical, physical, and biological) for the AgNPs production have been developed over time and they are usually based on the reduction in a silver salt, used as a source of Ag^+^ ions, in the presence of reducing agents and different conditions [19]. Although the use of chemical reagents is the most popular approach in the synthesis of AgNPs, the potential toxicity of different materials certainly limits the possibilities of their application in the biomedical field [20].

In this regard, the search for new strategies has led to the development of green methods for AgNPs synthesis. Jaiswal et al. synthesized AgNPs using lignin as a reducing and capping agent in the carrageenan matrix to prepare functional carrageenan-based hydrogels [21]. Yang et al. developed a Chitosan/*Bletilla striata* polysaccharide-composited microneedle with antibacterial and antibiofilm properties, using the catechol groups of tannic acid for the in situ formation of AgNPs [22]. Similarly, Li et al. formulated a sodium hyaluronate-graft dopamine hydrogel doped with AgNPs, produced in situ by exploiting the catechol groups of dopamine for the reduction in Ag^+^ [23].

Polymeric nanofibrillar scaffolds, due to their suitable physicochemical and morphological properties, represent excellent platforms for tissue engineering and biomedical applications [24,25,26]. The microstructure of such systems is comparable to the tissues extracellular matrix (ECM) and it is crucial for adhesion, growth, and cell proliferation [27,28]. Synthetic polymers, such as polyesters, have excellent advantages in terms of mechanical properties, biodegradability, biocompatibility, and low costs [29]. Polylactic acid (PLA) is among the most used polyester for its remarkable mechanical properties, slow degradation in vivo, and the absence of immune or inflammatory responses [30,31,32], which led to several applications in tissue engineering and in tendon [33], bone [34], skin [35], and cardiovascular regenerative medicine [36], and nanomedicine for cancer [37] and lung diseases treatment [38]. However, the absence of intrinsic antibacterial properties makes the PLA scaffolds susceptible, in vivo, to be colonized by microorganisms, increasing the risk of generating an antibiotic-resistant bacterial biofilm [39,40]. Different approaches have been adopted to overcome this limitation. Popleka et al. functionalized a PLA electrospun scaffold with ascorbic and fumaric acid, after plasma treatment, providing to the system antimicrobial activity against Gram-positive *Staphylococcus aureus* and Gram-negative *Escherichia coli* bacteria [41]. Martin et al. developed a 3D-printed PLA-based scaffold doped with minocycline for bone regeneration in order to overcome the typical infections associated with bone implants [33].

Furthermore, mixing PLA with polymers that reduce Ag^+^ and stabilize the AgNPs, advanced PLA scaffolds, with antimicrobial properties can be obtained. For example, polymers with catechol groups have an excellent ability to reduce Ag^+^ in situ, while stabilizing the AgNPs formed [23,42,43]. Xiang et al. used a mussel-inspired dopamine-based polymer (poly (carboxybetaine-co-dopamine methacrylamide)) for AgNPs in situ synthesis and capping. The obtained system was then immobilized onto amino-modified cotton gauze resulting in an antibacterial cotton dressing with excellent silver loading stability [42]. Zhang et al. developed a PLA-based fibrillar scaffold doped with polydopamine-coated gold nanoparticles exploiting the reducing ability of catechol on the structure of polydopamine to reduce Ag^+^ to AgNPs in situ [43].

In this work a nanofibrillar scaffold based on PLA, having antibacterial and antibiofilm properties, through the in situ formation of AgNPs, was developed. The use, in mixture with PLA, of a polymer bearing pendant catechol groups, via successive aminolysis reactions of polysuccinimide (PSI) with dopamine and ethanolamine, named poly(α,β-(N-(3,4-dihydroxyphenethyl)-L-aspartamide-co-α,β-N-(2-hydroxyethyl)-Laspartamide) (PDAEA) allowed us to combine the remarkable properties of PLA with the possibility to reduce silver in situ by green method.

The treatment with cold plasma at low pressure of N_2_ of the scaffolds, before the treatment with AgNO_3_ and formation of the AgNPs, increases the wettability of the scaffolds and subsequently allows to obtain a homogeneous formation and distribution of AgNPs in the fibrillar matrix. The advantage of this approach is related to the possibility to easily produce electrospun scaffolds with reproducible physicochemical and morphological properties and intrinsic reducing features conferred by the presence of PDAEA, without the need of using nanocomposite reducing materials, embedded in the fibers or anchored to the scaffold surface, that can negatively influence the fibers morphological features and the reproducibility of the production process. The obtained systems were characterized in order to evaluate their chemical–physical properties and antimicrobial potential. This has been tested in vitro against *Pseudomonas aeruginosa* which is a model of serious and difficult to treat nosocomial infections [44].

For this reason, PLA- and PDAEA-based scaffolds can represent good examples of antibacterial and antibiofilm systems in tissue engineering and regenerative medicine.

## 2. Results and Discussion

### 2.1. Synthesis and Characterization of PDAEA and PLA/PDAEA Electrospun Scaffolds

PDAEA (chemical structure and characterization reported in Figure S1) is highly functionalized with dopamine (more than 60 mol% with respect to the repetitive units) and so its presence can guarantee a high concentration of reducing moieties in the electrospun scaffold. This represents an advantage compared with the catechol-functionalized natural derived polymers, such as polysaccharides [10,45,46], that usually show degree of functionalization lower than 20 mol%. Moreover, dopamine contributes to confer a lipophilic character to the macromolecule making possible to obtain an electrospinnable blend with PLA in a mixture of organic solvents, even if the presence of PDAEA at the concentration used leads to the necessity to use a slightly higher voltage in the electrospinning process compared with that used for pristine PLA dispersion in order to generate a continuous and stable filament.

**Figure 1:**
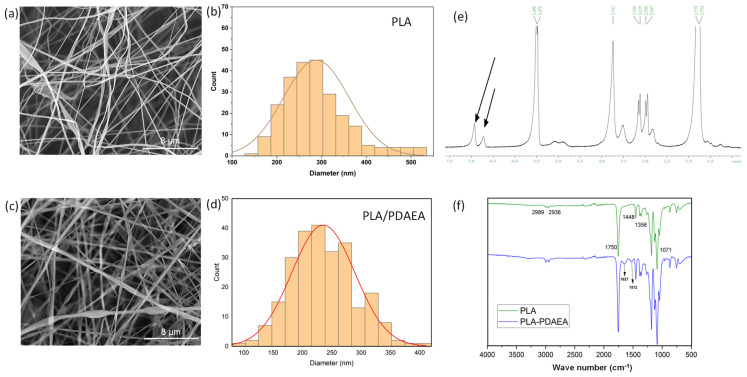
SEM images of electrospun scaffolds of PLA (**a**) and PLA/PDAEA (**c**). Size distribution of fibers diameter for scaffolds of PLA (**b**) and PLA/PDAEA (**d**). The ^1^H−NMR spectrum of PLA/PDAEA electrospun scaffold (**e**). FT−IR spectra of PLA and PLA/PDAEA scaffolds (**f**).

The morphological properties of the scaffolds were studied through SEM analyses, whose results are shown in Figure 1a,c where the fibers are randomly distributed. The diameter distribution analysis showed that the average diameter was about 288 nm for PLA fibers and 235 nm for PLA/PDAEA fibers (Figure 1b,d). This difference is probably related to the need to use higher voltages during the electrospinning process of PLA/PDAEA dispersion as reported in previous studies [47]. Furthermore, PLA/PDAEA fibers appear uniform, without deformation and quite similar to those of PLA, so the use of PDAEA does not represent a limitation in the electrospinning process.

Moreover, as shown in Figure 1e,f, spectroscopic analysis, carried out on the electrospun fibrillar scaffolds (dissolved in organic solvent), confirmed the presence of PDAEA. In particular, the ^1^H-NMR spectrum, obtained by dispersing the polymer in DMSO-d_6_ (Figure 1e) shows peaks at 6–7 ppm, which can be related to the three aromatic protons of dopamine present in PDAEA, as also observed in Figure S1b. Since the oxidation of dopamine results in a shift of these peaks, the ^1^H-NMR analysis allows to affirm that the dopamine of the PDAEA does not undergo oxidation during the electrospinning process.

The FT-IR spectrum of PLA/PDAEA (Figure 1f) scaffold, compared with that of PLA, shows the appearance of two enlarged peaks of modest intensity (1627, 1512 cm^−1^) related to the stretching of the two different amidic carbonyls, as also found in the FT-IR spectrum in Figure S1c.

### 2.2. Plasma Treatment of the Scaffolds

Although PLA scaffolds have several advantages in tissue engineering applications, it is often necessary to change their surface properties in order to overcome an important limitation related to the lipophilic nature of the polyester which leads to scaffolds’ low wettability. As already stated, the purpose of this work was to exploit PDAEA catechol groups to produce AgNPs onto the surfaces of the electrospun fibers. To obtain a homogeneous distribution of the in situ-produced metal nanoparticles it is essential that the fibrillar matrix can be effectively wetted by the silver salt solution. Since the PLA/PDAEA scaffold showed a water contact angle similar to that obtained of pristine PLA (about 120°), scaffolds were treated with cold plasma at low pressure of N_2_ in order to increase their wettability. It is reasonable to suppose that, because of the use of N_2_, the scaffold surface is functionalized with amine groups [48,49,50], which contributed to provide hydrophilic properties to the electrospun fibers, increasing their wettability.

After plasma treatment, no chromatic variations in scaffolds were observed, indicating that dopamine did not undergo oxidation reaction. Macroscopically, no morphological variations were observed.

### 2.3. SEM Analyses and Contact Angle Measurements on Plasma-Treated Scaffolds

**Figure 2:**
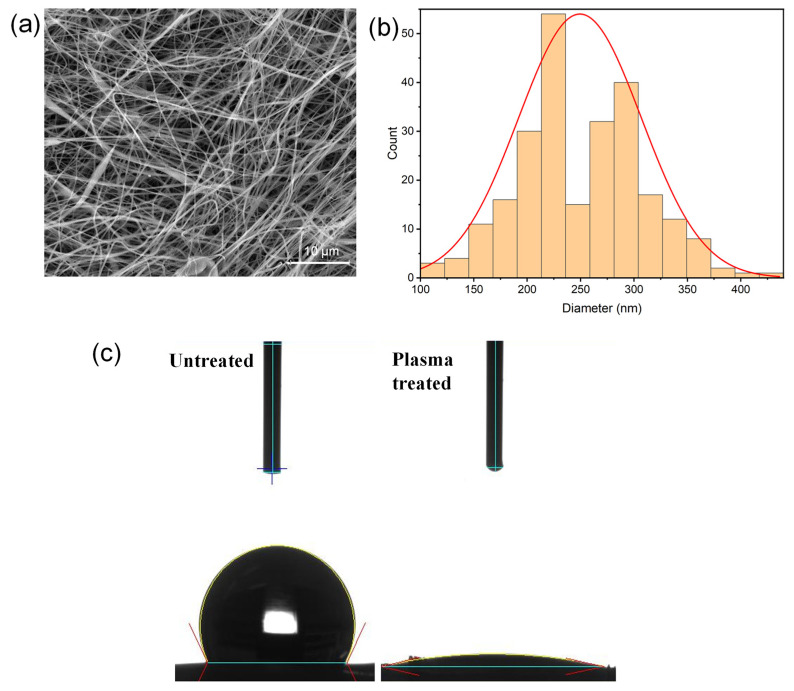
SEM images (**a**) and size distribution of fibers diameter (**b**) for PLA/PDAEA after plasma treatment. Contact angle measurement (**c**) before (left) and after plasma treatment of PLA/PDAEA scaffold (right).

Since plasma treatment can determine microscopic changes in the fibrillar structure of scaffolds, resulting in changes in physicochemical properties, SEM analyses were performed to investigate the morphological features of the scaffolds after the treatment. The results, reported in Figure 2a, shows how plasma-treated scaffold does not undergo microscopic alteration in the fibrillar structure being the fibers diameter distribution practically unchanged (PLA/PDAEA average diameter = 249 nm) compared with plasma-untreated scaffold (Figure 2b). 

The increased wettability was demonstrated by measuring the contact angle before and after plasma treatment as shown in Figure 2c. The obtained contact angle values are 121.94° and 1.88°, respectively: this means that plasma treatment increases the wettability of the scaffolds almost completely.

### 2.4. AgNPs In Situ Production

AgNPs were produced in situ exploiting the reducing capacities of dopamine through a simple and reproducible method, starting from AgNO_3_ solutions as previously reported by Li et al. [23]. The absence of additional reducing reagents has limited the possible formation of secondary and/or toxic products. 

**Figure 3:**
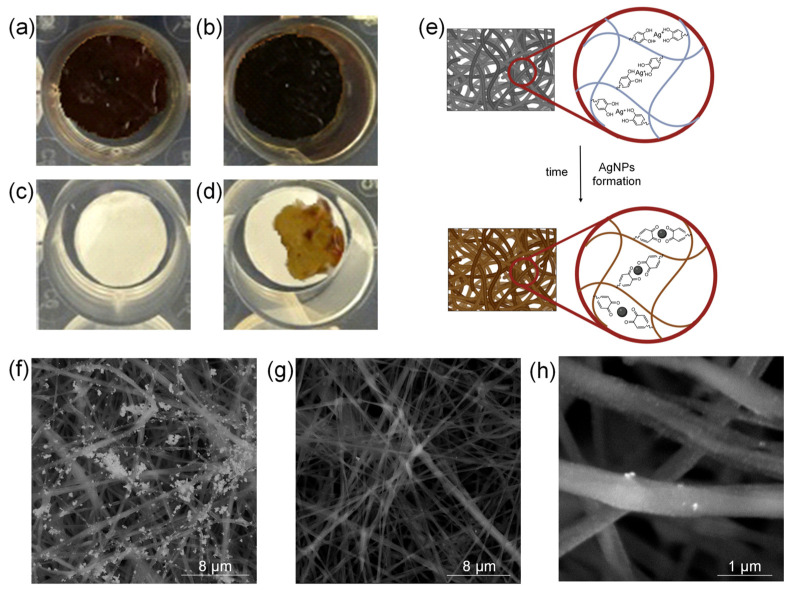
Pictures of plasma-treated PLA/PDAEA scaffolds after 72 h of incubation with: 30 mM AgNO_3_ solution (**a**), 100 mM AgNO_3_ solution (**b**) and bidistilled water (**c**). Picture of plasma-untreated PLA/PDAEA scaffold after 72 h of incubation with 100 mM AgNO_3_ (**d**). Schematic representation of AgNPs in situ formation (**e**). SEM images of plasma-untreated (**f**) and plasma-treated (**g**,**h**) PLA/PDAEA@AgNPs scaffolds.

As shown in Figure 3a,b, scaffolds color turned to a dark brown probably due to the dopamine oxidation and the AgNPs production. Figure 3e shows schematically the formation of AgNPs. In particular, the presence of catechol groups allows, firstly, the chelation of Ag^+^ ions and, subsequently, their reduction and anchoring onto the fibers surface. This likely avoids the burst release in the physiological milieu of the produced metallic nanoparticles [42].

It is interesting to notice that plasma-untreated scaffold (Figure 3d), because of its poor wettability, showed a chromatic variation only in the drop deposition point while, scaffolds incubated with water showed no colour variation (Figure 3c). This shows that plasma treatment is of great importance for obtaining samples with a homogeneous distribution of antibacterial nanoparticles.

To further corroborate the hypothesis that the formation of AgNPs is ascribable mainly to the presence of PDAEA, the PLA scaffold was used as control in the process of in situ nanoparticles production. Results in Figure S2b showed that after plasma treatment, only a light chromatic variation was observed for the scaffold after the incubation with AgNO_3_ solution. It is likely to suppose that both the free radical produced during the plasma treatment and free amine groups inserted onto the scaffold surface can confer to the PLA scaffold some mild reducing capacity that can lead to the formation of low amount of metal nanoparticles. 

### 2.5. PLA/PDAEA@AgNPs Scaffolds Morphological Characterization

SEM analyses were carried out on PLA/PDAEA@AgNPs samples to validate the macroscopical observation already described. As expected, the plasma-untreated sample, due to the lower wettability shows superficial coarse AgNPs aggregates (Figure 3f) that, on the contrary, are not observable onto the fibrillar structure of the plasma-treaded sample (Figure 3g). At higher magnification, SEM images of the plasma-treated PLA/PDAEA@AgNPs samples show nanometric bright spots homogeneously distributed onto the surface of the fibers and attributable to the colloidal metal nanoparticles (Figure 3h). No significative differences in the SEM analyses were detected from the PLA/PDAEA@AgNPs scaffolds incubated with the silver salt solution at two different concentrations. This a clear confirmation both of the effective AgNPs formation and of their retaining on the scaffold surface after the purification procedure.

### 2.6. PLA/PDAEA@AgNPs Scaffolds Surface Characterization

**Figure 4:**
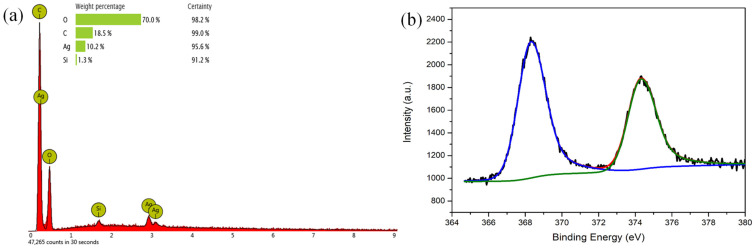
EDX (**a**) and XPS (**b**) analyses on PLA/PDAEA@AgNPs.

The presence of silver onto the surface of the PLA/PDAEA scaffolds treated with AgNO_3_ solution was once again confirmed by energy-dispersive X-ray spectroscopy (EDX) analysis, conducted concomitantly with SEM experiments (Figure 4a).

An X-ray photoelectron spectroscopy (XPS) analysis was also carried out to confirm that silver was present in its reduced form. In the spectrum shown in Figure 4b, it is possible to find the presence of two well-defined peaks at 368.2 eV and 374.2 eV, typical of the metallic silver (Ag 3d) [51,52]. The absence of further peaks attributable to different species shows that all the silver in the scaffold is in the form of metal nanoparticles. This confirms the effectiveness of both the method of preparation of the AgNPs and the washing procedures that allowed the removal of all Ag^+^ not reduced from the scaffold.

### 2.7. Ag Quantification on PLA/PDAEA@AgNPs Scaffolds

The quantitative analysis performed on the PLA/PDAEA@AgNPs shows no significant (confidence level = 95%) increasing concentration of Ag immobilized in the scaffolds on the rising of the concentration of the treating AgNO_3_ solution. The metal level on the scaffold is far higher than that of the starting solution due to the immobilization of the Ag on the small amount of the weighed scaffold polymer, which acts as a solid-phase concentrating media. Treating the scaffolds with AgNO_3_ 30 mM and 100 mM (incubated for 72 h) the following respective concentration of silver were determined: 8.7 × 10^4^ ± 3 × 10^3^ μg/g and 8.8 × 10^4^ ± 4 × 10^3^ μg/g.

The quantitative analysis performed on the mineralized PLA scaffolds by ESI-MS experiments showed the presence of Ag. However, the signal’s integral of the control sample was in the order of the Limit of Detection (LOD) and below the Limit of Quantitation (LOQ) of the analytical method.

### 2.8. In Vitro Cytocompatibility Studies

AgNPs can have a cytotoxic effect on eukaryotic cells [53,54]; for this reason, cytocompatibility studies were performed by indirect procedure, without putting the scaffolds in direct contact with the cells. Our main aim was to assess the possible release of large quantities of silver that can be toxic to cells, as reported in the literature.

Since it has been amply demonstrated that electrospun PLA scaffolds produced with a procedure similar to ours are highly biocompatible [41,55,56,57], the viability of the developed devices has been compared with that of PLA scaffolds.

**Figure 5:**
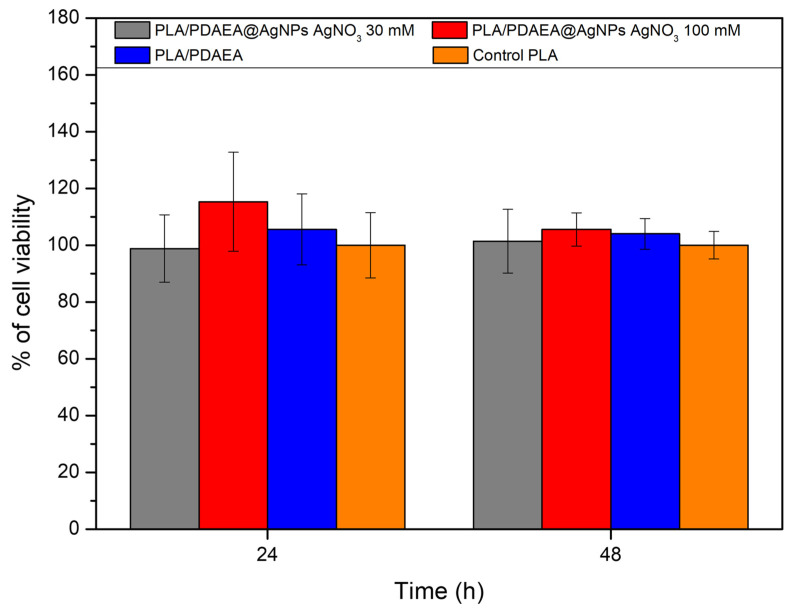
Results of MTS assay at two different times (24 h and 48 h) expressed as a percentage of cell viability compared with control PLA scaffolds.

As shown in Figure 5, there are no statistically significant differences between PLA/PDAEA@AgNPs scaffolds or PLA/PDAEA and control samples (plasma-treated PLA scaffolds) both after 24 h and 48 h of incubation (viability higher than 80% compared with the PLA control). This means that the presence of PDAEA and the exposure of cells to scaffolds containing AgNPs did not affect cell viability over time and that the PLA/PDAEA@AgNPs scaffolds do not release cytotoxic molecules or substances.

This is further corroborated by the increase in cell metabolic activity between the two-time point of analysis.

### 2.9. Inhibition of Bacterial Biofilm Formation of PLA/PDAEA@AgNPs Scaffolds

**Figure 6:**
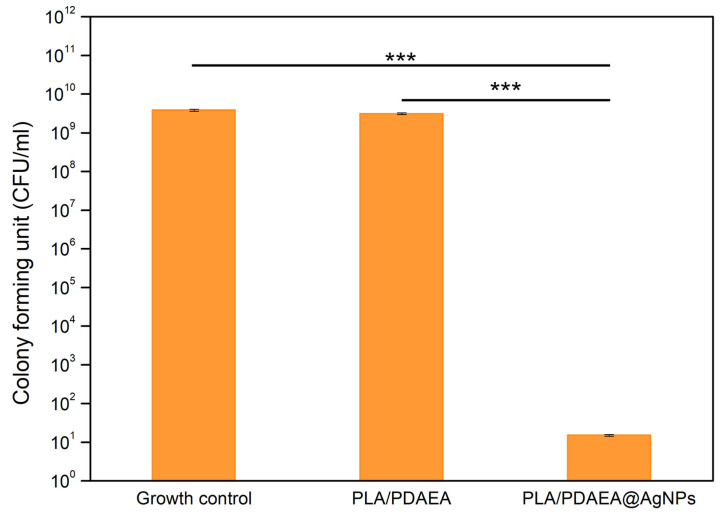
Antibiofilm activity against *P. aeruginosa* ATCC 15442 of PLA/PDAEA scaffolds prepared incorporating 30 mM AgNO_3_. Histograms show the CFU/mL of tested bacterial strain obtained by viable plate counts method after incubation at 37 °C for 24 h. Growth control bacterial culture without scaffold; PLA/PDAEA scaffold without AgNO_3_; PLA/PDAEA@AgNPs scaffold with 30 mM AgNO_3_. *** *p* < 0.001.

Since this type of device might be implanted in the medium/long term, they should possess not only optimal mechanical and physical properties but also an antibiofilm activity against commensal and/or pathogenic bacteria. AgNPs antibiofilm activity is well-known in the literature [11,58,59]. To demonstrate that the presence of AgNPs on the PLA/PDAEA scaffolds inhibits the bacterial adhesion and the consequent biofilm formation, the antibiofilm potential of PLA/PDAEA@AgNPs scaffolds against *P. aeruginosa* ATCC 15442 was evaluated (Figure 6). The growth control bacterial counts, corresponding to bacterial culture without scaffolds, were equal to 3.1 × 10^9^ CFU/mL, similarly the control scaffolds (PLA/PDAEA without AgNO_3_) were equal to 3.8 × 10^9^ CFU/mL, suggesting that the exposure to PLA/PDAEA scaffolds did not prevent the *P. aeruginosa* biofilm formation. In bacterial cultures samples, incubated with PLA/PDAEA@AgNPs, a drastic and statistically significant (*p* < 0.001) reduction in the viable bacteria count was observed up to 1.5 × 10^1^ CFU/mL, achieving a logarithmic reduction in 8.65 compared with control samples of PLA/PDAEA without AgNO_3_ or growth control. These results suggest the capacity of the PLA/PDAEA@AgNPs scaffolds to significantly interfere with pseudomonal adhesion, colonization, and biofilm formation.

Moreover, the inhibition of *P. aeruginosa* planktonic growth in presence of the PLA/PDAEA@AgNPs scaffolds was evaluated by measurement of the optical density at 600 nm (Table 1). The scaffold with AgNO_3_ showed a significant antimicrobial activity against planktonic strain. Optical density, measured after 24 h of incubation of *P. aeruginosa* with scaffold PLA/PDAEA treated with 30 mM AgNO_3_ resulted in drastically lower (significant reduction with *p* < 0.05) than growth control or growth in the presence of PLA/PDAEA scaffold without AgNPs.

The experiments were carried out to prove the effect of PLA/PDAEA@AgNPs scaffolds for the effective growth reduction in *P. aeruginosa* in planktonic and sessile conditions.

**Figure 7:**
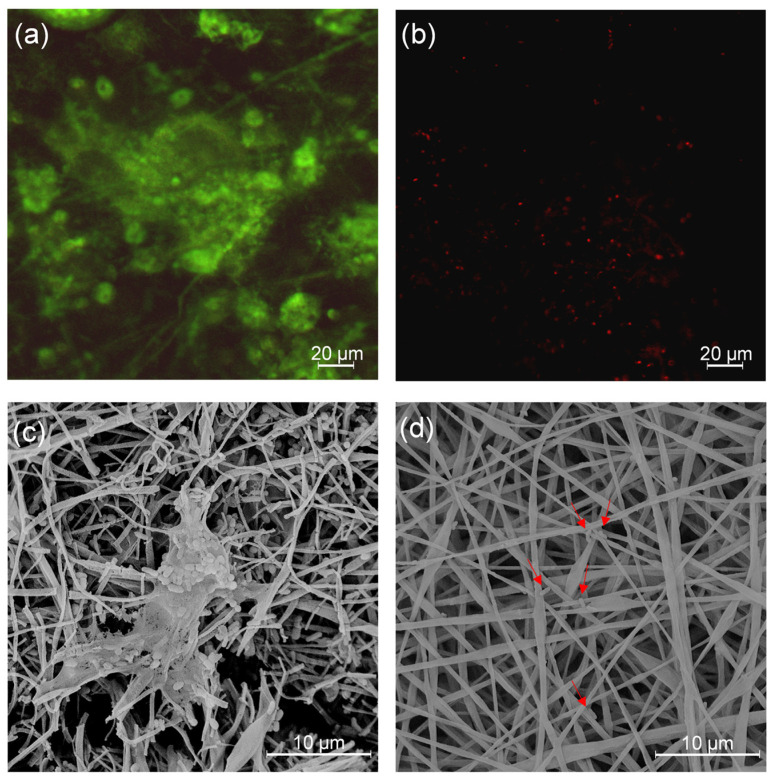
Results of CSLM analyses on PLA/PDAEA (**a**) and PLA/PDAEA@AgNPs 30 mM (**b**) scaffolds. SEM images of PLA/PDAEA (**c**) and PLA/PDAEA@AgNPs 30 mM (**d**) scaffolds after incubation with *P. aeruginosa* cultures. Red arrows indicate bacteria on AgNPs-doped scaffolds.

To observe the antibiofilm effect of the PLA/PDAEA@AgNPs scaffolds, the Live and Dead test (L&D) was conducted and confocal analyses were performed on samples incubated with bacterial cultures as described in paragraph 3.9. Results (Figure 7) confirmed that PLA/PDAEA scaffolds were colonized by *P. aeruginosa* (Figure 7a) while no significant colonization was detected in PLA/PDAEA@AgNPs scaffolds (Figure 7b). SEM analyses confirmed the trend of antibiofilm activity of PLA/PDAEA@AgNPs scaffolds. PLA/PDAEA scaffolds (Figure 7c) were colonized by *P. aeruginosa*, while only a few bacteria were observed on AgNPs-doped scaffolds (Figure 7d).

These results are comparable with others reported in the literature and suggest that similar scaffolds can be applied as antibiofilm-forming systems in regenerative medicine.

### 2.10. Antibiofilm Activity Evaluation

**Figure 8:**
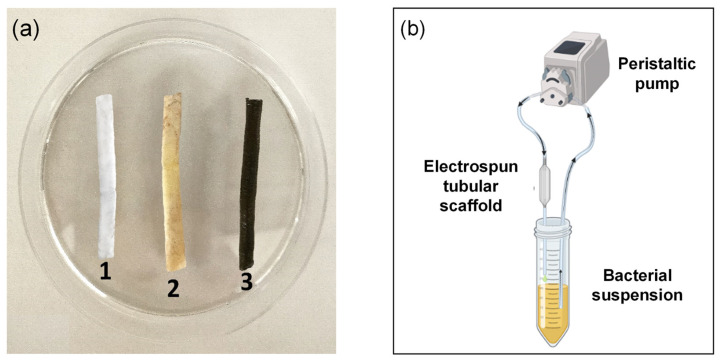
Electrospun tubular scaffolds (**a**) of PLA (1), PLA/PDAEA (2) and PLA/PDAEA@AgNPs (3). Schematic representation of the system used for the antibiofilm activity evaluation (**b**).

In order to evaluate the potential antibiofilm activity in “dynamic” conditions to mimic a potential situation in which an implantable device is subjected to a physiologic fluid flux and can be colonized by microorganisms (as example a catheter used for urological tissue engineering), tubular scaffolds (Figure 8a) were prepared and used as described in Figure 8b. In this case, a tubular PLA scaffold was also used to study the influence of PDAEA on the macroscopic mechanical performance of the sample. A bacterial suspension of *P. aeruginosa* was set up to flow continuously for 6 h and at room temperature inside the tubular scaffolds using a peristaltic pump.

Then, a L&D and SEM analyses were carried out to evaluate the possible formation of bacterial biofilms on the support. For the L&D test, the samples were firstly washed with DPBS and then treated with L&D solution according to the standard protocol. Results showed how the PLA scaffold was colonized by *P. aeruginosa* (Figure S3a). The same result can be found in the PLA/PDAEA control tube (Figure S3b). Instead, no significant colonization is detected unless some isolated clusters of non-biofilm-forming *P. aeruginosa* (Figure S3c) is in PLA/PDAEA@AgNPs tubular scaffold. The presence of AgNPs has significantly reduced the colonization of the inner wall of the scaffold, preventing the adhesion and the biofilm formation process.

**Figure 9:**
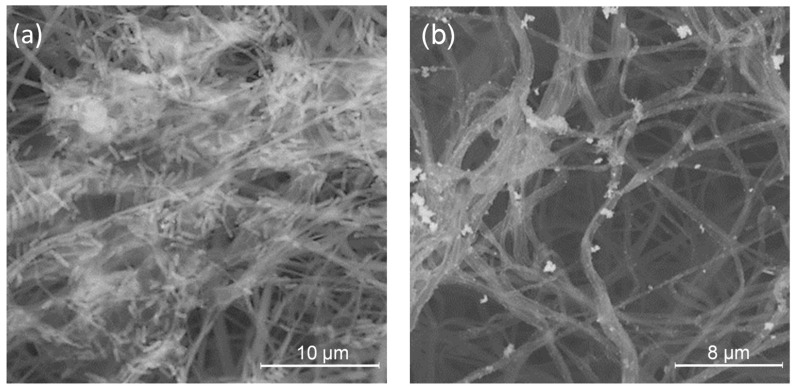
SEM images of PLA/PDAEA (**a**) and PLA/PDAEA@AgNPs (**b**) tubular scaffolds after antibiofilm treatment.

Furthermore, SEM analyses (Figure 9) confirmed L&D results: PLA (Figure S4) and PLA/PDAEA (Figure 9a) scaffolds were colonized by the bacteria forming biofilm. Instead, as we expected, no bacterial adhesion is observed in PLA/PDAEA@AgNPs scaffold (Figure 9b). The obtained results are significant and lead to a possible application of the developed systems in urological regenerative medicine.

## 3. Materials and Methods

### 3.1. Materials

Dimethylsulfoxide anhydrous (DMSOa), hexamethyldisilazane, hydrogen peroxide 30%, ethanol absolute, dopamine hydrochloride, dichloromethane (DCM), N,N-dimethylformamide (DMF), silver nitrate, and Dulbecco’s Phosphate Buffered Saline (DPBS) were purchased from Sigma Aldrich (Milan, Italy).

Nitric acid, ethanolamine (EA), and triethanolamine (TEA) were purchased from Fluka (Milan, Italy); Resomer^®^ R 202 H, Poly(D,L-lactide) (PLA) was purchased from Bidachem-Boehringer Ingelheim (Ingelheim, Germany); Dulbecco’s Minimum Essential Medium (DMEM), Live/Dead Cell Double Staining Kit, MC3T3-E1 (murine preosteoblastic cells) were purchased from Euroclone (Milan, Italy).

CellTiter 96^®^ AQueous One Solution Cell Proliferation Assay (MTS) was purchased from Promega (Milan, Italy).

### 3.2. Apparatus

The electrospinning process was conducted via a flow pump programmed KDScientific Mod. 78-8100INT (Milan, Italy), and a high voltage generator Spellman CZE1000R (Bochum, Germany). The rotating metal collector is custom type.

The ^1^H-NMR spectra were obtained with a Bruker Avance II 400 MHz spectrometer (Milan, Italy).

FT-IR spectra were obtained with Bruker Alpha in the wave number range of 400 and 4000 cm^−1^ (Milan, Italy). 

SEM and EDX analyses were performed with Phenom Pro X Desktop (Thermo Fisher Scientific, Rome, Italy).

The scaffold was mineralized by means of an automatic microwave digestion system CEM Discover SP-D 80 (CEM, Cologno al Serio, Italy). The quantification of silver ions was carried out using an HPLC Waters Alliance 2695 (Water S.p.a., Sesto San Giovanni, Italy) used as solvent delivery system coupled, by means a manual loop (Rheodyne 750, Milan, Italy) with hybrid mass spectrometer (Q-ToF Premier, Waters, Sesto San Giovanni, Italy). Mass spectra were recorded in positive ion mode using an electrospray ionization (ESI) source.

X-ray photoelectron spectrophotometer (XPS) analyses were conducted with the PHI 5000 VersaProbe II instrument (ULVAC-PHI, Inc., Kanagawa, Japan) with a source: Al Kα (1486.6 eV) and a 128-channel hemispherical analyzer, FAT mode.

Fluorescence images related to cytocompatibility tests were obtained with an AxioVert200 (Zeiss) microscope (Milan, Italy). Fluorescence images related to antibiofilm activity were obtained with an Olympus FV1200 confocal microscope (Segrate, Italy) with total internal reflection fluorescence (TIRF) integrated.

The scaffolds were treated with cold plasma equipment FEMTO B with nitrogen gas, low pressure (Diener, Ebhausen, Germany) coupled to a vacuum pump Pfeiffer PBF 71/2B-11RQ (Pfeifer Vacuum, Paderno Dugnano, Italy).

The contact angle on the scaffolds was measured using the Contact Angle 1000 C FTA instrument (First Ten Angstroms, Portsmouth, VA, USA).

Cell cultures were performed using an Eppendorf New Brunswik S41i incubator (Milan, Italy).

UV measurements were performed using an Eppendorf AF2200 spectrophotometer (Milan, Italy).

The assay for the evaluation of antibiofilm activity was conducted using a Laboratory Peristaltic Pump MINIPULSľ 3 Gilson (Milan, Italy).

### 3.3. Synthesis of PDAEA

Poly(α,β-(N-(3,4-dihydroxyphenethyl)-L-aspartamide-co-α,β-N-(2-hydroxyethyl)-Laspartamide) (PDAEA) was synthetized starting from polysuccinimide (PSI) [60,61,62,63], through successive PSI aminolysis reactions with dopamine and ethanolamine [64]. Initially 485 mg (5 mmol) of PSI were dispersed in 2 mL of DMSOa in a 25 mL flask, following argon bubbling for 15 min. Simultaneously, 948 mg of dopamine hydrochloride (5 mmol) was dissolved in 2.5 mL of DMSOa and subsequently an excess of TEA (2.5 mL) was added to remove the hydrochloride. The molar ratio between dopamine hydrochloride and repetitive units of PSI correspond to 1. The obtained mixture was degassed for 10 min and then added dropwise, under vigorous stirring, to the PSI dispersion. The reaction was carried out in the dark for 24 h at 60 °C under constant stirring. After 24 h the temperature reaction was reduced to 40 °C and 1.503 mL of ethanolamine (25 mmol) and the reaction was carried out for further 4.5 h. The molar ratio between ethanolamine and repetitive units of PSI was set to 5. The product was isolated by ethanol precipitation, centrifuged, and re-dispersed in DMSO (2 mL) three times. Then, the product was washed two more times in ethanol and recovered by vacuum-drying at room temperature with a yield of 130 % based on the PSI initial weight. The ^1^H-NMR and FT-IR analyses were performed on the obtained product. The degree of molar functionalization in dopamine (DD_DOPA_%) of PDAEA was calculated by ^1^H-NMR analysis. Figure S1a shows the pattern of the reaction of PSI aminolysis with dopamine and ethanolamine. The ^1^H-NMR and FT-IR analyses (Figure S1b,c) confirmed the successful reaction and purification of the product.

### 3.4. Production of PLA/PDAEA Electrospun Scaffold

Electrospinning dispersion was prepared as follows. PLA was dispersed in DCM at a concentration of 25% *w*/*v* while PDAEA was dispersed in DMF at a concentration of 10% *w*/*v*. The two dispersions were then mixed vigorously, using a vortex, in a ratio of 1:1 *v*/*v*, to obtain a stable dispersion, in which the weight ratio between PLA and PDAEA is 2.5:1. The obtained dispersion was then loaded into a syringe and electrospun through a metal needle with an internal diameter of 21 G, using 12 kV of voltage, a flow rate of 0.08 mL/min using a distance of 20 cm between the needle tip and the collector. The fibrillar scaffolds obtained were collected on an aluminum-coated rotating collector (diameter of 3.8 cm and rotation of 300 rpm). The ^1^H-NMR, FT-IR and SEM analyses were performed on the obtained scaffold.

A PLA control scaffold was electrospun as well from a dispersion prepared by mixing a dispersion of PLA in DCM (25% *w*/*v*) and DMF in a ratio of 1:1 *v*/*v*, using a voltage of 10 kV and the other same parameters. 

The tubular scaffolds used for the evaluation of the antibiofilm activity, were produced according to the same methods, but using a rotating metal collector with a diameter of about 0.4 mm.

SEM analyses and contact angle measurements were carried out on the PLA/PDAEA scaffolds.

### 3.5. Plasma Treatment of Scaffolds

Electrospun scaffolds were treated with cold plasma at low pressure of N_2_ using a Femto B system. Freeze-dried samples were treated for 10 min e with a power of 10% in a N_2_ saturated chamber (N_2_ flow = 15 mL/min, N_2_ pressure = 1–1.4 mbar).

After the plasma treatment, SEM analyses and contact angle measurements were carried out on the PLA/PDAEA scaffolds.

### 3.6. Production and Characterization of PLA/PDAEA@AgNPs Scaffolds

For the in situ synthesis of AgNPs, plasma-functionalized PLA/PDAEA scaffolds were treated with AgNO_3_ solutions (70 μL) at different concentrations (30 mM and 100 mM). Samples were incubated for 72 h under controlled humidity conditions at 37 °C and 5% CO_2_ atmosphere and then they were washed several times with bidistilled water to remove excess AgNO_3_ and freeze-dried.

In order to have control samples, two PLA scaffolds were treated with bidistilled water and other two with AgNO_3_ solution (30 mM, 70 μL). Moreover, PLA/PDAEA control samples were also prepared: scaffolds of PLA/PDAEA not-plasma-functionalized were treated with AgNO_3_ solution (30 mM, 70 μL) and scaffolds of PLA/PDAEA plasma-functionalized were treated with only bidistilled water. These samples were then incubated for 72 h, washed with bidistilled water and freeze-dried. SEM, EDX, and XPS analyses were performed on PLA/PDAEA@AgNPs scaffolds.

For the electrospun tubular scaffolds for the antibiofilm test, the treatment was performed by imbibing them with the 30 mM AgNO_3_ solution using a 24-h incubation time.

### 3.7. Ag Quantification on PLA/PDAEA@AgNPs Scaffolds

Solutions at different concentrations (0.05 mM, 0.1 mM, 0.2 mM, 0.5 mM, 1 mM, and 2 mM) in acidified water (1% nitric acid) were introduced into the mass spectrometer by a manual loop (10 μL). The HPLC was configured as simple solvent delivery system in isocratic mode (water with 0.1% formic acid) setting a solvent flow of 150 μL/min. The ESI experiments were carried out under the following experimental conditions: capillary voltage 3.5 kV, cone voltage 30 V, extraction cone 4.4 V, ion guide 1.2 V, source temperature 90 °C, desolvation temperature 300 °C, desolvation gas flow (N_2_) 530.0 L/h, cone gas flow 50.0 L/h, maintaining a mass range *m*/*z* 50–*m*/*z* 1000 and setting a scan time of 1 s and an interscan time of 0.1 s. The mass accuracy was always better than ±0.02 Th.

The ESI-MS spectra of silver-containing mineralized scaffolds were acquired under the same experimental conditions. 

Before mass spectrometry analysis, the PLA, PLA/PDAEA blank scaffolds, and PLA/PDAEA@AgNPs were digested and mineralized using a microwave oven with a high-pressure rotor. Each sample, carefully weighed, was treated with 1 mL of HNO_3_ (69%) and 1 mL of H_2_O_2_ (30%) and then mineralized at a temperature of 200 °C. After the digestion step, the clear, residue-free samples were made up to 2 mL of LC/MS grade water and analyzed.

The calibration curves were obtained using standard solutions at various concentrations of AgNO_3_ in an acidified water solution containing HNO_3_ at 1% *v*/*v*. A stock solution at 20.0 mM Ag^+^ concentration was prepared by weighting 0.340 g of AgNO_3_ and dissolving it in 100 mL of acidified water (HNO_3_ at 1% *v*/*v*). The following working standard solution at 2.00 mM, 1.00 mM, 0.500 mM, 0.200 mM, 0.100 mM, and 0.0500 mM were obtained by diluting the appropriate volumes of stock solution (5.00 mL, 2.50 mL, 1.25 mL, 0.500 mL, 0.250 mL, and 0.125 mL, respectively) in 50.00 mL flasks. Blank samples signals were acquired before sample analyses to determine the limits of detection (LOD) and quantification (LOQ) of the analytical determination. The linearity of the method (R^2^ = 0.997) was checked between 0.04 mM (LOD) and 2 mM and the LOQ (0.100) was significantly lower than the lowest concentrated sample analyzed (0.398 mM).

### 3.8. In Vitro Cytocompatibility Studies

In vitro cytocompatibility studies were performed on murine preosteoblastic cells (MC3T3-E1) by MTS colorimetric assay. The test was carried out on all scaffolds of PLA/PDAEA treated with AgNO_3_ (30 mM and 100 mM) and on control samples of PLA and PLA/PDAEA without AgNO_3_ treatment. Cells were cultured at 37 °C and 5% CO_2_ with a humidified atmosphere, in DMEM supplemented with 10% *v*/*v* of FBS, 1% *v*/*v* of penicillin–streptomycin solution, 1% *v*/*v* of glutamine solution, and 0.1% *v*/*v* amphotericin B solution. For the experiments, after trypsinization the cells were counted, re-suspended in DMEM, seeded into 24-well culture plates at a density of 7.0 × 10^5^ cells, and left to incubate overnight to allow adhesion. Then, after UV sterilization (λ = 250 nm, 125 W, 30 min for side), round-shaped scaffolds were fixed on plate-inserts which were placed in 24-well plates and immersed in 0.5 mL of DMEM. After 24 h and 48 h of incubation, cell viability was valued with MTS test according to the manufacturer’s specifications. The absorbance at 492 nm was read by a UV-vis spectrophotometer. Experiments were performed in triplicate. Statistical analysis for significance was conducted with the Student’s *t*-test; values with *p* < 0.05 were considered statistically significant.

### 3.9. Inhibition of Bacterial Biofilm Formation of PLA/PDAEA@AgNPs Scaffolds

One reference strain, *P. aeruginosa* ATCC 15442, was used in this study given its ability of producing a high amount of slime according to the methods and criteria proposed by Martorana et al. [11]. The viable plate counts method was used to quantify the total biofilm biomass formed on scaffolds of PLA/PDAEA treated with 30 mM AgNO_3_ compared with control samples of PLA and PLA/PDAEA without AgNO_3_. Biofilm formation on the surface of scaffolds was obtained in 24-well polystyrene plates fitting one disc for each well. The scaffolds were immersed in 2 mL of Tryptic Soy Broth (TSB) solution (supplemented with glucose 2 %) and 100 μL of bacterial suspension were added at the concentration of 1 × 10^6^ CFU/mL, prepared in NaCl 0.9% *w*/*v* [65]. Plates were then immediately incubated at 37 °C for 24 h. At the end of the incubation period, the bacterial suspension was removed using a micro-pipette, and the microbial growth in solution was evaluated by measurement of the optical density at 600 nm. The total biofilm biomass formed on scaffolds was removed with a sterile loop after washing with sterile NaCl 0.9% to remove non-adherent cells. Therefore, the biofilm formed on scaffolds was transferred in tubes with 10 mL of NaCl (0.9% *w*/*v* solution) and sonicated for 3 min. For each sample, 10-fold were prepared and 100 μL aliquots of each dilution were plated onto Tryptic Soy Agar (TSA) (Sigma Aldrich, USA) plates, followed by incubation at 37 °C overnight [66]. To quantify the number of viable bacteria in each system, the value of CFU/mL was determined, and the antibacterial activity was also detected in terms of logarithmic (log) reduction. Log reduction was calculated by subtracting the difference between the log CFU/mL of the growth control (scaffold without AgNO_3_) and the log CFU/mL of PLA/PDAEA@AgNPs scaffolds. Statistical analysis for significance was conducted with the Student’s *t*-test; values with *p* < 0.05 were considered statistically significant.

To observe the effect of the PLA/PDAEA@AgNPs scaffolds on biofilm, microscopy experiments were also performed. The bacterial cultures in plates with scaffolds of *P. aeruginosa* were prepared as before mentioned. After 24 h of incubation the scaffolds were washed, stained (L&D), and observed through a confocal laser scanning microscope (CSLM) [9]. SEM analyses were also carried out on the same samples; after drying as reported in paragraph 3.8, the samples were mounted on aluminum stubs, vacuum-coated with a 10 nm thick layer of gold (Sputter Coater LuxorAu, Luxor Tech, Nazareth, Belgium), and then observed by SEM.

### 3.10. Antibiofilm Activity Tests

To assess the antibiofilm properties, tubular scaffolds (ø = 4 mm) of PLA/PDAEA@AgNPs, PLA/PDAEA and PLA were treated with a suspension of *P. aeruginosa* ATCC 15442.

The bacterial suspension was prepared, inoculating one loopful from a culture, grown at 37 °C for 24 h on TSA, into TSB containing 2% *v*/*v* glucose. The bacterial culture was incubated overnight at 37 °C. After incubation time, the bacterial suspension of *P. aeruginosa*, containing ~1 × 10^6^ CFU/mL, was moved continuously inside tubular scaffolds for 6 h, using a Laboratory Peristaltic Pump MINIPULSľ 3 Gilson. Then, samples were cut longitudinally to expose the inner wall coming into contact with the bacterial dispersion, washed with sterile DPBS and SEM analyses and L&D tests were carried out. For L&D, the samples were treated with 500 μL of solution prepared following the standard protocol, incubated for 30 min at 37 °C and then images were taken with an AxioVert200 fluorescence microscope [9,10].

For SEM analyses, the samples were treated with a 4% formaldehyde solution in DPBS for 30 min at 37 °C. Then the scaffolds were dried by washing with hydroalcoholic solutions with increasing ratio ethanol/water (30:70, 50:50, 70:30, and 100:0) for 10 min and finally with hexamethyldisilazane for 15 min, removing the solvent at the end of washing to allow the samples to air dry. The samples were analyzed by SEM.

## 4. Conclusions

Electrospun scaffolds with non-specific antimicrobial and antibiofilm properties were developed. AgNPs in situ synthesis by exploiting the catechol groups of PDAEA was successfully carried out and represent a valid alternative method for the development of AgNPs-doped scaffolds. Plasma treatment of electrospun scaffolds before adding AgNO_3_ solution, allowed to obtain an uniform distribution of AgNPs. XPS analyses confirmed the presence of only metallic silver, which indicates the goodness of the synthetic process. Cytocompatibility studies showed that the AgNPs-doped scaffolds do not have cytotoxic effects on murine preosteoblastic cells and do not affect cell viability over time. In vitro antibacterial and antibiofilm activity evaluation, against clinically important pathogen *P. aeruginosa*, shows good capability to prevent biofilm formation which is particularly challenging to be treated by using conventional antibiotics. Eventually, PLA/PDAEA@AgNPs scaffolds showed interesting results in preventing *P. aeruginosa* biofilm formation, suggesting their possible use in regenerative medicine.

## Figures and Tables

**Table 1:** Mean values and standard deviation (SD) of optical density of *P. aeruginosa* after 24 h of incubation in presence of PLA/PDAEA scaffolds with or without 30 mM AgNO_3_ compared with control cultures (without scaffolds). PLA/PDAEA scaffold without AgNO_3_; PLA/PDAEA@AgNPs 30 mM scaffold with 30 mM AgNO_3_.

Planktonic Growth	Optical Density Mean Values ± SD
Growth control	0.391 (±0.049)
PLA/PDAEA	0.403 (±0.013)
PLA/PDAEA@AgNPs	0.079 (±0.007)

## Data Availability

Not applicable.

## Supplementary Materials

The following supporting information can be downloaded at: https://www.mdpi.com/article/10.3390/ijms232315378/s1.
