# Supplementary material for: Developing Antibiofilm Fibrillar Scaffold with Intrinsic Capacity to Produce Silver Nanoparticles
Source: Int J Mol Sci. 2022 Dec 6;23(23):15378. doi: 10.3390/ijms232315378 (PMC9737318; doi:10.3390/ijms232315378)
Supplement: Supplementary file 1 [file ijms-23-15378-s001.zip › ijms-2045661-supplementary.pdf]

Developing antibiofilm fibrillar scaffold with intrinsic capacity to produce silver nanoparticles

Supplementary materials

1. Synthesis and characterization of PDAEA

PDAEA, whose structure is reported in Figure 1 have been synthesized and purified as reported in literature[1]. Figure S1a shows the pattern of the reaction of PSI aminolysis with dopamine and ethanolamine. The first reaction step allowed the opening of a part of the PSI rings with dopamine. Despite the use of an excess of catecholamine compared to the repetitive units of the PSI, the steric encumbrance given by the benzene ring of dopamine itself, inhibits the quantitative opening of all rings. This effect obviously becomes more marked the more the polymer backbone is functionalized. After 24 hours, an excess of ethanolamine was added by attaching all remaining PSI rings not reacted with dopamine, leading to their opening.

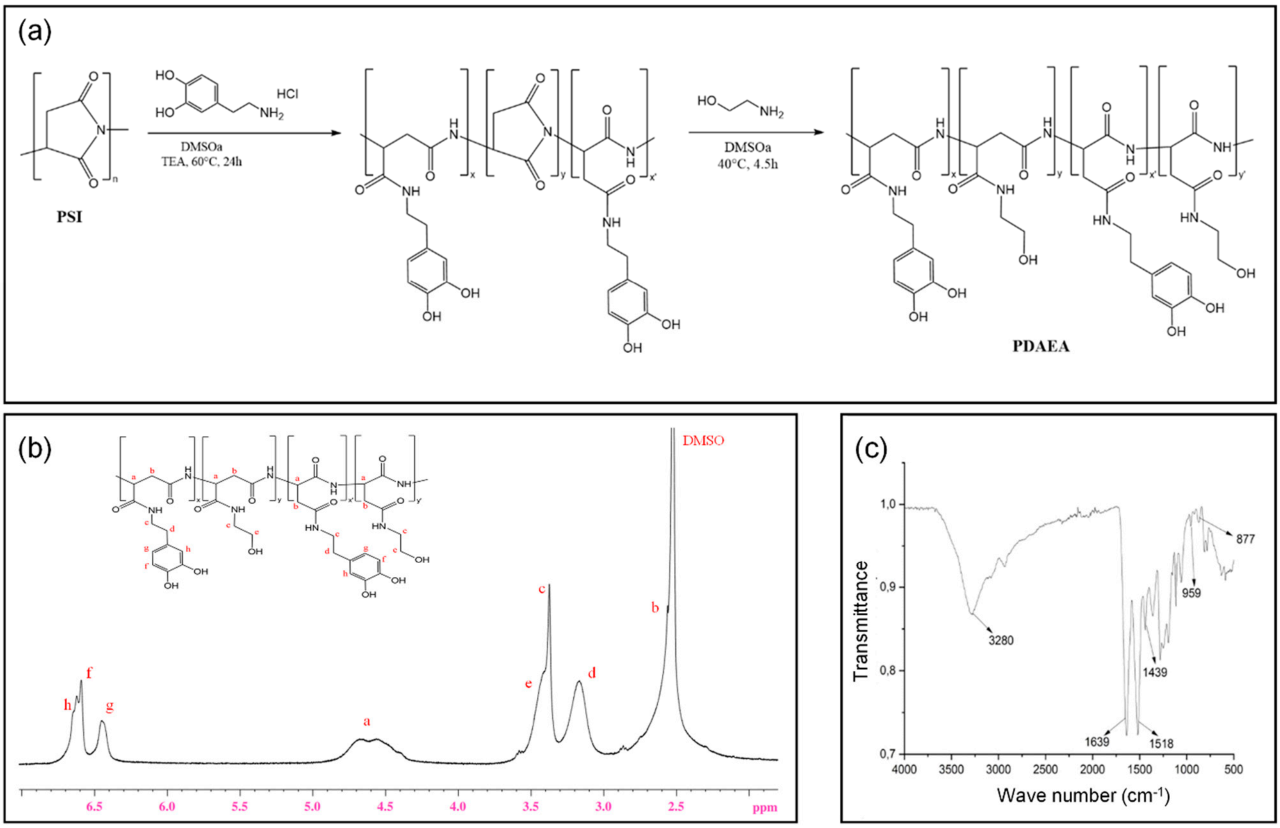

Figure S1. Schematic representation of PDAEA synthesis (a). 1H-NMR (b) and FT-IR (c) spectra of PDAEA.

PDAEA structure have been confirmed with 1H-NMR (figure S1b) and FT-IR analysis (figure S1c). In 1H-NMR spectrum, the disappearance of the peaks at 5.27 ppm of the -CH- proton of the PSI and the appearance of that at 4.4-4.8 ppm, attributable to the methyne proton of the PDAEA (Figure S1b, letter a), indicates that the opening reaction of the succinimidic rings of the PSI was quantitative. The three peaks h, f, g around 6.5 ppm are related to the 3 aromatic protons of dopamine. Peak c (3.3-3.5 ppm) is relative to -CH2- protons in α to the amino group of the polyaspartamide side chain for both the dopamine replaced and ethanolamine substituted portions. Finally, the peak e (3.5-3.6 ppm) is attributed to the CH2- in α to the primary hydroxyl groups of ethanolamine chain. Comparing the area of peak a and peak d (3.0-3.4 ppm, relative to methylene protons of the polyaspartamide lateral chain replaced by dopamine), the derivation degree in dopamine is approximately 67,6 mol% compared to PDAEA repetitive units.
Moreover FT-IR analysis was performed (figure S1c). As expected, PDAEA spectrum shows the disappearance of typical absorption bands at 1796 and 1710 cm-1 of PSI (symmetrical and asymmetric stretching of the amidic

carbonyl group of the succinimide ring) and the appearance of picks at 1639 and 1518 cm-1 relating to the stretching of the two different amide carbonyls: the former is related to the opening of PSI ring with dopamine and the latter is related to the opening with ethanolamine; this shows that the reaction was quantitative. The widening of band at 3280 cm-1 is due to the presence of OH stretching of both dopamine and ethanolamine. The bands at 1439, 959 and 877 cm^{-1} give further confirmation of the presence of the benzene ring as they can be attributed to the symmetrical and asymmetric aromatic C-C stretching.

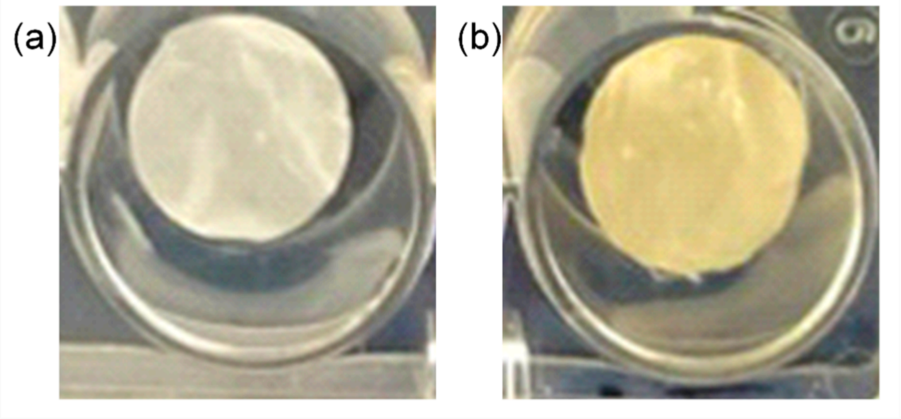

Figure S2. Plasma functionalized PLA scaffold treated with water (a). Plasma functionalized PLA scaffold treated with 30mM AgNO3 solution (b).

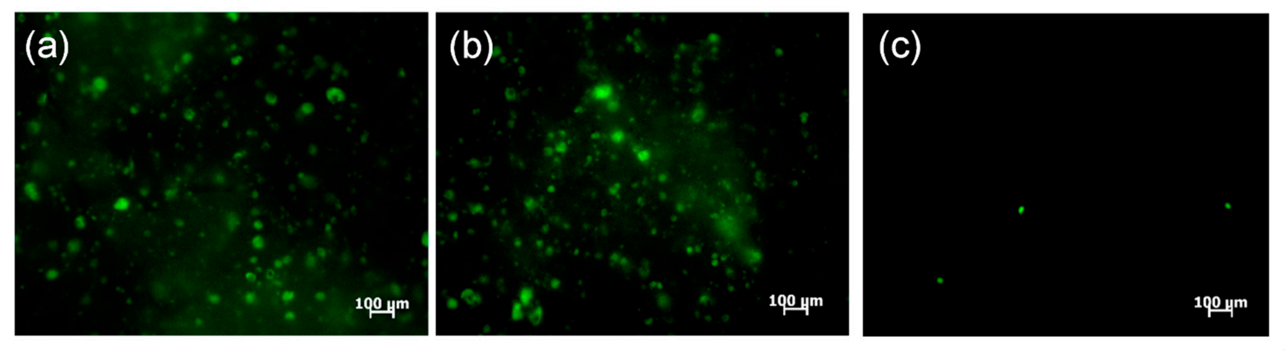

Figure S3. Results of Live&Dead test assay of P. aeruginosa growth on PLA (a), PLA/PDAEA (b) and PLA/PDAEA@AgNPs (b) tubular scaffolds. Images show viable bacterial cells.

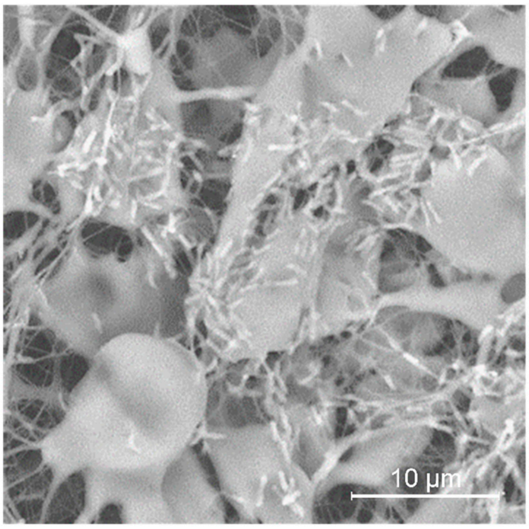

Figure S4. SEM images of PLA tubular scaffolds after antibiofilm activity evaluation assay.

References

1. Gong, C.; Lu, C.; Li, B.; Shan, M.; Wu, G. Injectable dopamine-modified poly(α,β-aspartic acid) nanocomposite hydrogel as bioadhesive drug delivery system. *J. Biomed. Mater. Res. Part A* **2017**, *105*, 1000–1008, doi:10.1002/JBM.A.35931.
